# The high-profile case as ‘fire object’: Following the Marianne Vaatstra murder case through the media

**DOI:** 10.1177/1741659017718036

**Published:** 2017-08-28

**Authors:** Lisette Jong, Amade M’charek

**Affiliations:** University of Amsterdam, Netherlands; University of Amsterdam, Netherlands

**Keywords:** Fire object, high-profile case, murder case, STS, trial by media

## Abstract

In 1999 a girl named Marianne Vaatstra was found murdered in a rural area in the Netherlands. In 2012 the perpetrator was arrested. Throughout this period as well as thereafter, the Vaatstra case was never far removed from media attention and public debate. How did this murder become such a high-profile case? In this article we employ the concept of the ‘fire object’ to examine the high-profileness of the Vaatstra case. Law and Singleton’s fire metaphor helps to attend to objects as patterns of presences and absences. In the Vaatstra case it is in particular the unknown suspect that figures as a generative absence that brings to presence different versions of the case and allows them to proliferate. In this article we present four different versions of the Vaatstra case that were presented in the media and which shaped the identities of concerned actors. The unruly topology of fire objects, we argue, might well explain the high-profileness of such criminal cases.

## Introduction

On the morning of 1 May 1999, friends and family of 16-year old Marianne Vaatstra from Zwaagwesteinde, a small village in the northern Dutch province of Frisia, went searching for her. She had not returned home after spending Friday night partying in the nearby village of Kollum. They found her mutilated dead body in a rural meadow situated in between Kollum and Zwaagwesteinde. On the following Monday, newspapers reported that police investigations had indicated that Marianne had been raped before she was murdered and that the perpetrator had slit her throat with a knife. Some 13 years later, on 18 November 2012, local farmer Jasper S was arrested upon a DNA match found through a type of DNA dragnet called familial searching. He confessed, and was convicted of murder and sexual assault in 2013.

Meanwhile, not a year went by when the ‘Vaatstra case’, as it became known, was not attended to in Dutch print and broadcast media. In 1999, the number of articles mentioning the case in national newspapers ranged from 12 to 70 per source, while the regional *Leeuwarder Courant* published 131 articles mentioning ‘Marianne Vaatstra’. When Jasper S was arrested and convicted, the case received even more media attention than in the year of the murder. The Vaatstra case had become a high-profile case, well-known within and even beyond the Netherlands.^1^ As a district attorney stated in a 2001 documentary: ‘[The Vaatstra murder] has become a national case … in which something is apparently at stake for everyone’.^2^ A 2009 newspaper interview with Marianne’s relatives similarly noted that ‘Marianne seems [to be] of everyone’ (*Leeuwarder Courant*, 25 April 2009). How is it that the murder of a girl in a rural province came to engage so many?

There is a scholarship on high-profile cases (e.g. Chancer, 2005; Cottle, 2005; Innes, 2003, 2004; Soothill et al., 2002, 2004) involving different approaches. Here two of these works, one taking a more quantitative and the other a more qualitative approach, will be briefly discussed. Criminologists Soothill and colleagues (2002, 2004) differentiate between ‘mega’, ‘mezzo’ and ‘routine’ cases in terms of the amount of press coverage certain homicides receive in a given period of time. Mega cases are understood in terms of intrinsic qualities, such as having a ‘stranger murderer’ or ‘multiple dead bodies’. While the characteristics of mega cases differ, they are all rather ‘unusual’ compared to other homicide cases, which make them particularly interesting (Soothill et al., 2002). Following the reporting trajectories of 13 identified mega cases in the British *Times*, Soothill and colleagues (2004) suggest that mega cases follow a trajectory connected to both the process of the criminal justice system and case-related ‘incidents’ that generate peaks in media attention. However, the reporting trajectories of high-profile cases that become entangled with ‘wider societal agendas’ turned out to be rather unpredictable (Soothill et al., 2004: 1). Sociologist Chancer studied how certain high-profile cases, an analytical subcategory she calls ‘provocative assaults’, become ‘vehicles for crystallizing, debating, and attempting to resolve contemporary social problems’ (Chancer, 2005: 5). In particular, she attends to how these cases are politically mobilized to address concerns regarding structural inequalities of gender, race and class. Chancer identifies a dualistic framework in media reporting and public debate aligned with the prosecution and defence positions that, sometimes problematically, forces people to choose sides.

The analytical notions briefly discussed above, the *mega case* and the *provocative assault*, are interesting and have fed into our analysis of the Vaatstra case. However, we struggled with the fact that these perspectives either took the reported *case as a given* object with set characteristics that can be counted, and/or focused on *representations* and meanings that are constructed within a framework of pre-existing social structures. Either way, whether the focus is on the quantity or quality of representation, the reported case, as an object, is taken for granted or left unattended. The case itself is then assumed to be a singular, underlying event to which meanings can be attached. By contrast, our point of departure in this article is not how the case is *represented* in the media but how the case, and in particular its high-profileness, is *done*. Inspired by Science and Technology Studies (STS), especially material-semiotic approaches, we follow the case through the media. Doing so, we unravel how the case itself takes different shapes throughout the investigation process. Taking the Vaatstra case as a shape-changing object, rather than a singular event, enables us to attend to how through enacting different versions of the case various worlds and practices that seem disparate and unrelated are drawn together to become the heart of the matter of the case. In this article we therefore ask: what kind of object is the high-profile case? How may an answer to this question contribute to understanding its high-profileness? Answering these questions, we also aim to demonstrate the relevance of an STS approach to media analysis.

It may come as no surprise that murders are rather messy, and so are high-profile cases. Indeed, they qualify as ‘messy objects’; ‘objects that cannot be narrated from a single location’ (Law and Singleton, 2005: 348). More specifically, building on our extensive qualitative analysis of the Vaatstra case through the media, we argue that such high-profile cases can be made comprehensible in terms of Law and Singleton’s notion of the *fire object*.

## STS and the high-profile case as object

The interdisciplinary approach taken in this article builds on work in the field of Science and Technology Studies (STS), and material semiotics in particular. One of the concerns of STS has been how objects get assembled and how they assume a quasi-universal or natural form. On the face of it, objects seem to pass as matters of fact, thereby obscuring their process of making. Yet, throughout their existence, objects are dependent on the very practices that helped produce them. These practices help to hold them together as such (e.g. Latour, 1987). This is the famous Actor Network approach. This approach suggests that objects are not singular entities but configurations of various different entities held together in material-semiotic relations (Law, 2004). For example, a DNA profile cannot be reduced to nature or to the DNA of an individual. It *folds* within itself the DNA of others to whom it has been compared, the technology used to produce the profile, the visual technology used to read it, the statistical models used to analyse the results, the theory of evolution and of DNA mutation rates, as well as legal regulation, police expertise and so on and so forth (M’charek, 2014). They all contribute to the stability of the DNA profile as an object (M’charek, 2016). This *irreducibility* of objects (Latour, 1993) also means that the division between the scientific and the social, between nature and society becomes problematized (Barad, 2007; Foucault, 1980; Haraway, 1988, 1991; Latour, 1991). This problematization is not merely epistemological (how to get a theoretical handle on a given object) but ‘ontological’ (how does an object come about) (Law, 2004; M’charek, 2013; Mol, 2002; Puig de la Bellacasa, 2011: 87). The relational ontology that underpins much work in STS implies that things do not pre-exist their relating. As relating is considered an activity, these practices become the object of study.

STS scholars have suggested different metaphors to understand the normativities of objects. Metaphors of the network, fluid, fire and folded object have been introduced to not only understand how objects are shaped but also how they change as they move across practices (Callon, 1986; Law and Mol, 2001; Law and Singleton, 2005; M’charek, 2014; Mol and Law, 1994). In this article we argue that the high-profileness of the Vaatstra case resonates with Law and Singleton’s (2005) notion of the ‘fire object’. Law and Singleton employ the metaphor of fire to analyze the ruptures and discontinuities between the different versions of an object – in their case, alcoholic liver disease. They suggest that these different versions nevertheless hang together as a pattern of presences and absences: ‘Fire-like objects … are generated in juxtaposition with realities that are necessarily absent, even though they bring versions of those realities to presence’ (Law and Singleton, 2005: 345). In the Vaatstra case it is notably the unknown perpetrator who figures as the generative absence, as the other who is not there but whose absence makes different versions of the case proliferate.

The versions of the Vaatstra case that we analyze below bring to presence realities of senseless violence, safety at night, xenophobia and forensic DNA, while at the same time transforming those discontinuous realities. We also analyze how these versions relate to other local issues as well as (inter)national concerns, co-constituting the spaces in which the Vaatstra case becomes a high-profile case.

The first section attends to how the Vaatstra murder became a case of *senseless violence*. As such, the case spoke to a national concern that was entangled with a politics of belonging in which Marianne’s life became a particularly grievable one. The second part addresses how the fact that Marianne was murdered along a dark cycle path was used by villagers to focus attention on the vulnerability of cyclists in rural areas. It thus became a case of *safety at night*. The third section examines how uncertainty about the identity of the unknown perpetrator translated into accusing ‘an asylum seeker’ of the murder. Linked to debates on national asylum policy, the case became one of *xenophobia* in the Netherlands. The final version of the case discussed in this article attends to the Vaatstra murder as a case of *forensic DNA*. It addresses the ways debates about new and promissory technologies were entangled with developments in the Vaatstra case. These versions drew in local, national and international audiences as they were fuelled by, but also provided fuel for lingering societal concerns. Our argument is that these processes through which the case kept changing shape and content made for the high-profileness of the case. Before we elaborate on the versions outlined here, first a note on the process of data collection and analysis.

## Data and analysis

**Figure 1.**
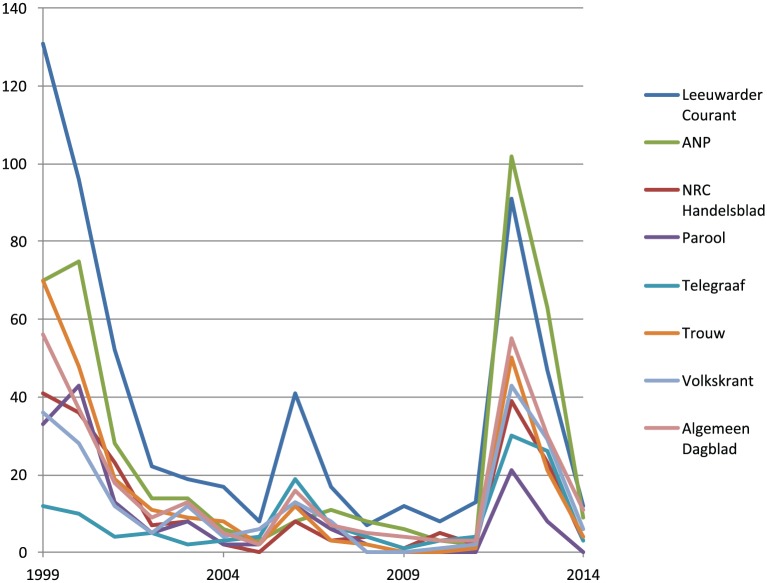
Amount of articles per year that mention the Vaatstra case for 8 news sources over the 1999–2014 period.

The dataset used in this study consists of all articles that were available through the LexisNexis database that mention ‘Marianne Vaatstra’ in 12 national and two Frisian regional Dutch newspapers and several Dutch news and opinion magazines, spanning a period from May 1999 to December 2014. This searching strategy enabled the methodology of following the case through the media and accordingly attending to its public reality beyond the course of the criminal justice system. Alongside the news articles, we included broadcasts of ‘crime watch’ shows, news programmes and documentaries that were available online. The eventual database comprised 2844 newspaper articles and 24 broadcasts. Figure 1 shows the flow of news articles that mention on the Vaatstra case for eight news sources for which data were available over the full 1999–2014 period.

We archived and analyzed the news articles and broadcasting notes using the qualitative data analysis software ATLAS.ti. A chronological overview and periodization was made and a thematic analysis conducted. In order to deal with the large amount of data, the local *Leeuwarder Courant* was chosen as the initial source to work from. Searching this newspaper generated the most hits for our search terms. More importantly, local newspapers address local concerns and events that may not appear in national newspapers or that are not reported on in detail. As such, newspapers partake in the activity of scale-making. This focus enabled us to take into account how the murder came to matter locally not only through accusations and suspicion towards local asylum seekers, as was also widely attended to in the national media, but also through the notion of ‘senseless violence’ and the ‘unsafety’ of unlit cycle paths. Interestingly, the *Leeuwarder Courant* does not shy away from publishing quotes from interviews and written materials in the local Frisian language, even when the main body of the text is in Dutch. For the purpose of this article, however, all data have been translated into English. Where specificities of the Dutch or Frisian terms matter, we elaborate on the translation in the notes section.

Other newspapers and broadcasting data were woven into the analysis so as to triangulate, elaborate on key themes, check references and understand translations made between different media outlets. Contrasting sources was also relevant to become aware of what was not reported on.

## A case of ‘senseless violence’

In the late 1990s, public debate in the Netherlands was concerned with what was referred to as the increase in ‘senseless violence’.^3^ The Dutch term ‘senseless violence’ (*zinloos geweld*) was coined by the district chief of the mid-Frisian police in 1997 to characterize the murder of a young Frisian man who had died after getting into a physical fight on his way home from a night out in the provincial town of Leeuwarden. This case became a reference point that mobilized media attention for cases that were ever since and retrospectively, irrespective of their great diversity (De Haan, 2011: 37), clustered as cases of senseless violence (*Leeuwarder Courant*, 15 September, 1997; Pouwels and Vegter, 2002).

Senseless violence was not a judicial category. However, in 1999 the Ministry of Justice came up with a definition of senseless violence that characterized cases of spontaneous and incidental acts of ‘intentional violence’ against a ‘randomly selected victim’ (Ministerie van Justitie, 1999). Although the suspect and his motivations remained unknown, qualifying the Vaatstra murder as one of senseless violence enacted the case as such and shaped the expression of emotions and moral outrage. On the Friday following the murder, friends of Marianne organized a ‘silent march’^4^ attended by thousands of people from across the Netherlands. The silent march figured as an event that was not only an act of commemoration and mourning for the victim, but also a protest against senseless violence. The silent march helped to enact the Vaatstra murder as a case of senseless violence and made it part of a national concern.

### A grievable life

Marianne’s name was added to a list of names on a long banner carried during the ritual march on the annual National Day Against Violence. Marianne thereby became part of a category of victims of senseless violence. Newspapers reporting on these events reiterated the case as such. In Butler’s (2009) terms, the names on the banner could be considered a collection of particularly ‘grievable lives’, lives that became eligible for public mourning. They were presented as innocent victims, recognizable human lives, stressing the similarities between them and the majority of Dutch society. To be sure, what came to matter in the identity of these victims was their ‘likeness’ (Butler, 2009), in the double sense of the word. Marianne was accordingly presented in the media as a nice schoolgoing daughter of Frisian parents, who worked in the local grocery store on the weekends and regularly enjoyed the nightlife in nearby villages. A Frisian family expressed this familiarity as follows: ‘It could have been one of our children. It is such a terrible thing. We should do something about it together’ (*Leeuwarder Courant*, 3 May 1999). The ‘it’, the senselessness of this violence, enacted the Vaatstra murder as a societal problem that affected not just the victim and her relatives but a broader ‘we’. Caring for Marianne as a victim of senseless violence thus implicated a politics of belonging (Mepschen, 2016). This reality, however, depended on absences, things that were othered and did not come to matter as senseless violence.

The asylum seeker centre near Kollum and its residents had been under attack ever since a vocal group of villagers had begun insinuating that the murderer was to be found there. In October 2000, a resident of the centre was stabbed with a knife by two young men when he was on his way home from the train station. The asylum seekers organized a protest march in which the stabbing came to stand for the ongoing violence and hatred they had been confronted with since the Vaatstra murder. While the protestors emphasized the particularity of the stabbing, the director of the centre claimed that it was just a case of ‘bad luck’; the victim could ‘just as easily have been a Frisian’ (*Leeuwarder Courant*, 10 October 2000). In the newspaper, the offenders were identified as a bunch of ‘troublemakers’ who would stab ‘anyone’ at the slightest provocation. Here the articulation of the selection of the victim as random and the act as incidental did not lead to a qualification of the stabbing as a case of senseless violence. Nor was the stabbing allowed to matter as a racist crime, as the particularities of the context were denied and the identity of the victim accordingly kept from mattering. He was made into an ‘anyone’; a not so grievable, othered life.

### Nightlife violence

In the late 1990s, senseless violence was popularly associated with an apparent increase in ‘nightlife violence’. In the media, the concern with nightlife violence was particularly present in the form of parental worries about teenage children. A woman who, after the Vaatstra murder, called on Dutch society to hang the flag at half-mast with a black mourning ribbon to protest against ‘nightlife violence’ was explicitly identified as a parent of a teenage son (*Leeuwarder Courant*, 4 May 1999). A year later, in response to the murder, a group of ‘mothers’ launched an awareness campaign on Frisian nightlife and established the foundation Against Senseless Violence Northeast Frisia (*Leeuwarder Courant*, 8 July 1999).

But there was also something related to this notion of nightlife violence working as an *absent presence* that shaped the Vaatstra case (Law, 2004). Following the incident in 1997, the Leeuwarden police investigated nightlife violence in the city, leading to the publication of the report ‘Committed to nightlife’ (Bunk et al., 1998). The report was discussed in the *Leeuwarder Courant.* The reasons for the increase in violence and conflict, so it was reported, were the use of alcohol and the observation that going out had increasingly become a group activity. Conflicts between groups were attributed to the differences between them, understood in terms of class and different ‘cultural backgrounds’ (*Leeuwarder Courant*, 24 June 1998). In a later article, this conclusion was also exemplified in terms of gendered relations: ‘A remarkable conclusion of the report “Committed to nightlife” was that nightlife violence is often caused by “intolerance and discrimination” between groups of allochtones and autochtones.^5^ “Deviant cultural beliefs lead for example to non-conforming ways of approaching women”’ (*Leeuwarder Courant*, 5 January 1999).

When located in Frisian nightlife through the police report and the Frisian media, the notion of senseless violence became differentially specified. The implied opposition between groups of ‘allochtones’ and ‘autochtones’ resonated with another story related to the murder. Marianne had supposedly been ‘threatened’ in a bar a few weeks before she was murdered (*Algemeen Dagblad*, 22 May 1999; *Leeuwarder Courant*, 5 May 1999). As it was narrated, she and her friends had gotten into a dispute with a group of teenage residents of the asylum seeker centre in a local bar. One of the boys had told Marianne to ‘shut up’ and had made a throat-slitting gesture while doing so. Although it was suggested that this particular boy would have been ‘very stupid’ to have actually done so, reiterating this account in the media fuelled suspicions towards asylum seekers in general.

As Law and Singleton (2005) teach us, fire objects are patterns of absences and presences. The enactment of the Vaatstra murder as a case of senseless violence depended on processes of othering; for example, processes in which certain lives were made less grievable or certain classes of people were made threatening. As we have shown above, the violence against asylum seekers did not come to qualify as an act of senseless violence. In addition, while the conclusion of the ‘Committed to nightlife report’ contributed to the culturalization of difference and conflict, the notion of senseless violence enacted a common belonging^6^ and obscured instances of racism. The implied politics of belonging fuelled the engagement of a nationwide public and enacted the Vaatstra case as a *case of senseless violence*, thereby contributing to its high-profileness.

## Safety at night: Dark cycle paths and the demand for road lighting

Following articles in the *Leeuwarder Courant*, the demand for road lighting had been an issue in the rural area before the murder. After the Vaatstra murder, the issue was reinvigorated. Safety and unsafety were not so much located in behaviour or physical bodies but in the physical environment. In newspaper articles that discussed the road lighting issue it was explained that Marianne was murdered ‘next to the *dark cycle path* along the Keningswei near Veenklooster’ (e.g. *Leeuwarder Courant*, 19 June 1999; *Leeuwarder Courant*, 5 July 1999; italics added). Tying their worries about dark cycle paths to the murder, a group of villagers united under the slogan ‘Light Gives Sight’. They collected thousands of signatures in a petition demanding illuminated cycle paths which they presented to the councillors of the municipalities of Kollumerland and Achtkarspelen in June 1999. After struggling with the costs, Kollumerland eventually met the demand but Achtkarspelen did not. This resulted in some main cycle paths between villages being lit only halfway (*Leeuwarder Courant*, 30 October 2001).

The demand for road lighting resonated with a form of self-organization that was explicated in news articles which addressed the nightly bike ride of young people from the rural villages surrounding the crime scene. Cycling home alone at night time was rather unusual for girls: if they did not take a taxi or were not picked up by parents, young people made sure to cycle in small groups (*Telegraaf*, 22 May 1999). This informal rule of conduct was seemingly broken by Marianne as she had presumably been alone on the night she was murdered. According to the accounts of the boys who had accompanied her halfway, Marianne had insisted on cycling the last kilometres by herself. Apart from disapproving commentary that they *shouldn’t* have let her go, it was by some believed that this *couldn’t* have happened because Marianne ‘wasn’t the kind of girl to do that’.^7^ Among them was Marianne’s father who doubted in particular the statement of one of the boys, Marianne’s boyfriend at the time, whom he held partly accountable for the death of his daughter (*Leeuwarder Courant*, 28 April 2000).

### Peter R de Vries

The doubt surrounding the boyfriend’s statement was reinforced in 2003 when crime reporter Peter R de Vries attended to the issue in his popular crime watch show on Dutch television. Although harshly criticized, De Vries emerged as a ‘super hero’ for the Vaatstra family whose members had publicly declared their loss of faith in the criminal justice system in August 1999. As Reijnders (2005: 646) describes: ‘where the police cannot or will not act De Vries steps in and comes to the aid of innocent victims’.

The concern with the dark cycle path disappeared from the media until it reappeared in a different guise in 2012 when Peter R de Vries dedicated an entire episode to the Vaatstra case. Things had changed and the crime reporter had now joined forces with the police. The show was given the opportunity to present the most recent offender profile and investigative technologies to the public. While the statement by Marianne’s boyfriend was no longer doubted, it served as a clue that Marianne may have known her killer and had maybe even planned to meet him that night. In the 2012 episode, the dark cycle path was enroled^8^ to reinforce this scenario. Through a reconstruction of the crime scene, the investigators concluded that the gender of a random passerby could not be read from a distance due to the darkness. It was suggested that this made it less likely for Marianne to have become the random female victim of a well-organized male lust murderer and more likely that she had a planned ‘rendez-vous’ or casual encounter and might have known the murderer. The notion of the perpetrator as a well-prepared rapist, ‘a predator waiting for his prey’, was accordingly overturned. He was now presented as an ‘occasional offender’, who had a friendly encounter with Marianne before he had possibly ‘flipped’.

Fire objects, as Law and Singleton (2005) have it, are characterized by jumps and discontinuities, that are nevertheless linked with each other. Above we have encountered the Vaatstra case as a case of senseless violence, while here it has evolved into a case about the safety and vulnerability of cyclists in rural areas. The dark cycle path has been a recurring site of interest that contributed to very different investigative scenarios of the case, enacting Marianne as either an innocent girl or as the girl who broke the rules by cycling alone in the dark and got into trouble. Yet, the importance of road lighting went beyond the criminal investigation process as it connected to a common concern about road safety. Acknowledging the vulnerability of young people cycling home at night in rural areas, it was suggested to introduce a night bus, more specifically a ‘disco bus’, to ensure safe transportation to and from the bars in the villages (*Telegraaf*, 22 May 1999). The Vaatstra case was thus enacted as a *case of safety at night*. This version, just like that of senseless violence, added to the high-profileness of the case.

## Xenophobia: An asylum seeker centre in the village

Most of the turmoil surrounding the Vaatstra murder in society and media outlets concerned the incrimination of asylum seekers by a vocal part of the local village population. Marianne was murdered in the proximity of a centre for asylum seekers, a temporary shelter at a former camping site. The centre became the locus of political antagonism as it was to be moved to a permanent location within the same village.

### Racism and policy critique

Soon after the murder, the municipality of Kollumerland decided to build a new permanent asylum seeker centre in the village. This was met with dismay by the villagers, and several groups assembled to protest the plans.^9^ The Vaatstra case played a pivotal role in the protest. ‘Is the decision on the establishment of the asylum seeker centre dependent on the nationality of the perpetrator?’, asked the mayor of Kollumerland rhetorically (*Leeuwarder Courant*, 16 July 1999). The public announcement in July 1999 that two former residents of the asylum seeker centre were potential suspects fuelled the anger in the area, culminating in a rally during the public information meeting in October 1999 about the new centre. Young men pelted the mayor with eggs. In a speech by the spokesperson of one of the protest groups, the centre was derogatively referred to as a ‘hotbed of criminal activities’ (*NRC Handelsblad*, 15 October 1999). Elsewhere in Frisia, the Vaatstra murder was also invoked to counter plans for new asylum seeker centres. In Lemmer, for example, a letter from concerned parents to the local government stated that the presence of an asylum seeker centre would pose a ‘sexual danger’ to their children (*Leeuwarder Courant*, 5 October 1999).

The local and national news media wrote mostly in understanding of the protesting villagers’ sentiments (see also Rigter, 2002: 37). The State Secretary of Justice addressed the ‘racist exclamations’ in a radio interview: ‘I understand the emotions, but I don’t want to condone [these exclamations]’ (*Leeuwarder Courant*, 18 October 1999). More critical voices argued that the murder simply served as a conduit for the slumbering xenophobia of the village population. In these debates, the identity of the protesting Frisians also became at stake. Were they blunt racists acting out of prejudice, or were they good citizens pointing out flaws in Dutch asylum policy?

The people in the Westerein read newspapers and watch television. They also know that there is something extensively wrong with the asylum policy in the Netherlands. They also know that the increasing dissatisfaction with that policy does not emanate from racism. (*Leeuwarder Courant*, 10 August 1999)

The Vaatstra murder thus became entangled with objections to national immigration policy as well as distrust of local actors such as the municipalities and the Frisian Public Prosecutor – especially since the asylum seeker centre was initially not taken into account by the investigation team, but also given the plans of the municipal council to build the permanent centre.

### Manner of death

The proximity of the asylum centre to the crime scene and the fact that Marianne’s throat was slit with a knife added to the suspicion placed on the centre’s residents. Slitting the throat was qualified as a ‘non-western’ way of killing, something that ‘a Frisian’ would never do (e.g. *Parool*, 27 June 2001; *Trouw*, 16 October 1999). Moreover, the slitting of throats was only a few times qualified as an Islamic practice of ritual slaughter, suggesting that it was self-evidently a characteristic of a potentially dangerous Other, and thus required no further explanation in the media. Accordingly, the late populist politician Pim Fortuyn^10^ reiterated this as ‘a reasonable thought’ in his widely read and well-cited column in the magazine *Elsevier* (16 October 1999). In line with one of his major campaign issues, Fortuyn continued the piece with a denunciation of Dutch asylum policy.

### Trial by media

In August 1999, on his crime watch show, Peter R de Vries showed the pictures and full names of the two former residents of the asylum seeker centre.^11^ The fact that the two men, one marked as suspect and the other as witness, had ‘disappeared’ from the centre on the day of the murder seemed to substantiate and legitimize earlier rumours. Although the Public Prosecutor stressed that one of them, Ali H, was marked as a suspect and not the perpetrator, in media reporting he was already convicted of the crime. The incrimination of Ali H thus enfolded as ‘trial by media’.^12^ He was addressed as a ‘fugitive’; the fact that he wasn’t considered a suspect from the outset was criticized as a ‘failure’ on the part of the police. When Ali H was arrested in Istanbul in October 1999, it was insinuated in the *Leeuwarder Courant* that his place of residence confirmed his presumed guilt:

He did not pick another European country where his fingerprints would have been filed in the police computers … He must have travelled over land on a false passport. Those are easily attainable through criminal organizations that transit people to Canada through asylum seeker centers. (*Leeuwarder Courant*, 14 October 1999)

Ali H was thus ‘guilty until proven innocent’ (Greer and McLaughlin, 2012: 4).

Ali H was exonerated when his DNA profile did not match the DNA of the traces found at the crime scene. Nevertheless, Ali H continued to figure in the media as the suspect in the Vaatstra case. In 2007 and 2010 it was repeatedly suggested that the man arrested in 1999 was ‘not the right Ali’. The idea that an asylum seeker had murdered Marianne never disappeared from the media. The indeterminacy of the figure of the unknown suspect translated into the incrimination of a generalized Other. In a documentary interview in 2003, the father of Marianne Vaatstra explicated the ongoing incrimination of asylum seekers as follows: ‘If our daughter’s murderer is not going to be caught, never is going to be caught, it will always be an asylum seeker to everyone, and to us as well’ (*IKON*, 2003).

The indeterminacy and othering had different effects that contributed to the high-profileness of the case. While the arrest of Ali H raised questions about the role of the media in the criminal justice system, his mediatized figure was generative of ongoing suspicion vis a vis this group. In a 2001 documentary on the Vaatstra case, the Public Prosecutor confessed that the arrest of Ali H in October 1999 was made under pressure from ‘public opinion’ while he was no longer a suspect for the investigation team. This fuelled public debate on the influence of the media in criminal investigations, a matter that was eventually debated in Parliament on the basis of the Marianne Vaatstra case.

Fire objects are energetic objects. They shift and change as they feed off things and practices that are othered. While depending on othering, the very otherness is generative. The Dutch asylum and immigration policy had been a matter of concern ever since the 1990s. This national concern translated locally into anxieties about e.g. the inhabitants of the asylum seeker centre. However, in this process the xenophobia and racism was actively denounced in the media. The problem was not with the local population not wanting to be in the proximity of ‘other people’, but with structural flaws in national policy. Presenting the Vaatstra case as an example of failed national immigration policy obscured the othering and criminalization of asylum seekers. Yet the xenophobia and racism was never completely silenced and kept resurfacing at the national and local level. We want to stress that there was not a linear trickling down of sentiments from the national to the local. The qualification of the manner of death as a non-Dutch way of killing in fact went the other way around. The complicated patterns of absence presences in this instance of the case helped to enact the Vaatstra case as a case of xenophobia and contributed to its high-profileness.

## Forensic DNA

Forensic DNA played a key role in the Vaatstra case. The course of the case and the development of Dutch legislation on the use of forensic DNA technologies in criminal justice had changed shape in close relation to one another.^13^ In search of the suspect, various forensic DNA technologies were introduced and legally regulated.

With the arrest of Ali H, a total of 25 DNA profiles of individuals had been compared to the biological evidence found on the victim. After his exoneration, the style of reporting shifted from ‘trial by media’ to highlighting the uncertainties surrounding the unknown perpetrator. The Public Prosecutor was quoted stating that at this point ‘it could have been anyone’ (*Leeuwarder Courant*, 16 October 1999). Upon this, cast as a ‘last resort’ by journalists, but ‘a powerful weapon’ by the police, in December 1999 a DNA dragnet was announced (*Leeuwarder Courant*, 8 January 2000). This was only the second time that this forensic method had been applied in a criminal case in the Netherlands, and it attracted a lot of media attention. While the dragnet did not lead to the perpetrator, it did provoke political debates about the forensic technology involved.^14^ In parliament and public discourse the Vaatstra case functioned both as a reference case to stress the need for further legal regulation of forensic DNA technologies and as an exemplary case in which the technologies had served the community, (non)suspects and the criminal investigation.

### A genetic suspect profile

In June 2000, the public prosecutor dealing with the Vaatstra case claimed that ‘the DNA profile of the perpetrator is still the most powerful weapon we have’ (*Trouw*, 14 June 2000). In the same month, a new suspect profile was presented in which the forensic DNA was differently engaged. The forensic laboratory in Leiden had been asked by the Public Prosecutor to infer the geographic descent of the unknown suspect based on the DNA. The inference of personal characteristics from DNA in the criminal justice process was then still unlawful, so the space in which this happened was simultaneously other to and part of the Vaatstra murder as a criminal justice case. In 2003 these boundaries were reconfigured when the ‘law on externally visible personal characteristics’ went into effect to regulate the use of this forensic genetic technology (M’charek, 2008).

Based on a study of the DNA, the forensic laboratory suggested that the offender was most likely a man of northwestern European descent. At about the same time, the results of the analysis of six behavioural experts were made public. They concluded that the perpetrator most likely lived within a radius of 15 kilometres from the crime scene. The results of the two expert studies got merged in newspaper headings reading: ‘Murderer Marianne is white [male] in the vicinity’ (e.g. *Algemeen Dagblad*, 14 June 2000; *Leeuwarder Courant*, 13 June 2000; *Trouw*, 14 June 2000). The implicated translations; northwestern European became ‘white’ and a radius of 15 kilometres became ‘vicinity’,^15^ both articulated closeness to the victim. However ‘clear’ and ‘powerful’ the DNA profile may have been, M’charek points out that the alleged ‘Dutchness did not help narrow the task of the criminal investigators’ (M’charek, 2008: 525–526) as it directed attention towards a majority instead of minority population. But it seemed that the primary purpose of the DNA analysis was to alleviate the social tensions surrounding the incrimination of asylum seekers (De Knijff, 2006). Based on the new suspect profile, it was perhaps unlikely that the perpetrator was an asylum seeker. But this did not stop the discriminatory and violent acts against asylum seekers in Kollum or the circulation of scenarios in which asylum seekers were accused of the crime.^16^

### Familial searching

In 2007, Marianne’s father, informed by an expert from a private forensic services company, had already pushed for ‘Y-chromosomal research’, thereby meaning familial searching (*Leeuwarder Courant*, 30 April 2007). Familial searching indicates a method where DNA comparison is not primarily aimed at finding a match, but at finding a relative of a possible suspect (Murphy, 2010). At the time this was not legally possible. In February 2012, however, an article in the *Leeuwarder Courant* headed ‘New DNA method [brings] hope in Vaatstra case’ announced that when the law regulating familial searching in criminal investigations was implemented later that year, the Vaatstra case would be the first case in which the method was going to be applied. In April, the search for near matches with the offender DNA profile in the DNA databank commenced. When this did not lead to any cues, a familial DNA mass screening was announced in which 8080 men were requested to participate. The familial searching technology was explicated to be particularly suitable in the Vaatstra case. The trace left by the perpetrator was qualified in relation to the technology as being of ‘top quality’ (*Leeuwarder Courant*, 18 February 2012) and the village community at stake as ‘geographically stable’^17^ and ‘in solidarity’ (*Leeuwarder Courant*, 19 November 2012). More than ever before, the media also played on the notion that ‘everyone in the region is suddenly a potential suspect’ (*Leeuwarder Courant*, 26 May 2012).

By the end of 2012, Jasper S was identified as the suspect based on a full DNA match, and in March 2013 he was convicted for the rape and murder of Marianne Vaatstra. The Vaatstra case ended up being a test case for the governance and legal regulation of forensic DNA in the Netherlands. Over the years, the unknown identity of the suspect continued to be the reason to take the next step in implementing novel technologies so as to learn about the suspect’s physical appearances and geographical descent as well as his familial kinship relations. Present day Dutch forensic DNA practice cannot be thought about outside of the Vaatstra case (M’charek, 2008; Toom, 2011). The entanglement of the case with advancement in technologies and regulations changed the nature of the case and made it into a *forensic DNA case* and thus contributed to its high-profileness. Notably, the generative absence presence in this version of the case is the figure of the unknown suspect of whom there was nothing more known than a ‘top quality’ biological trace.

## Conclusion

… fires are energetic and transformative, and depend on difference – for instance between (absent) fuel or cinders and (present) flame. Fire objects, then, depend on otherness, and that otherness is generative. (Law and Singleton, 2005: 344)

Examining the high-profileness of the Marianne Vaatstra murder case, this article engaged with senseless violence, safety at night, xenophobia and forensic DNA. We have argued that the Vaatstra case came in these versions and that the very capacity of the case to assume these different identities contributed to its high-profileness. The case engaged different local and national concerns as diverse as road lighting, a discobus, state of the art forensic DNA technology, nightlife violence among youth, national migration policy and racism against asylum seekers. It thus kept changing shape and rearing its head in the media. Law and Singleton propose a topology of fire to render such messy and uncontrollable objects comprehensible. A fire object ‘lives in and through the juxtaposition of uncontrollable and generative otherness’ (Law and Singleton, 2005: 347). The figure of the unknown suspect was one such generative absence, contributing to other absences, such as processes through which certain lives became grievable while others were not, or the constant racism that was hidden in the manner of death, i.e. the slitting of throats.

To be sure, the unknown perpetrator was not a stable figure but took different shapes in the versions presented here. The incidental senseless violence offender was other to the investigator’s lust murderer, and the Iraqi or Afghani asylum seeker yet again other to the white, northwestern European suspect generated by the genetic and behavioural expertise. The one could not transform gradually into the other, as they depended on specific versions of the case and presupposed other measures to be taken. But at the same time, the genetic suspect partially depended on the racist violence in the village community and the assumption that the suspect was an asylum seeker. To reiterate Law and Singleton (2005: 347), the:

versions are other to each other; they cannot be included in each other. At the same time (and this is the difficulty and the complication), they are also necessarily related to one another because they are part of the same [case] and interact with one another.

The case as a version of senseless violence slowly burned out but left its marks in the poem inscribed on the Marianne Vaatstra monument in Zwaagwesteinde. The versions that incriminated asylum seekers left a path of destruction. The permanent centre was never built and the one in proximity to the crime scene was closed in 2003. However, versions of the case in which an (generalized) asylum seeker figured as the perpetrator continued to spread uncontrollably. Even after the arrest of Jasper S, the figure of Ali H as perpetrator continued to be recreated in conspiracy theories. The case as a milestone in the regulation of forensic DNA has become fairly stable and durable. Not only did it translate into legislation of familial searching and the inference of visible characteristics based on DNA, it has also become the most dominant version of the case nationally and internationally – the case as a forensic DNA success story.

As the *Leeuwarder Courant* wrote, with the arrest and conviction of Jasper S, the villagers ‘woke up in a new reality … the perpetrator in the sensational Marianne Vaatstra murder case comes from their midst’ (*Leeuwarder Courant*, 20 November 2012). Forensic DNA technology had generated Jasper S as the perpetrator. He confessed to the rape and murder of Marianne and was convicted in 2013. The media exploded over his arrest and trial. It is this unusual and constant abundance of reporting in the media that we took as a generous gesture to reflect upon the phenomenon of the high-profile case. Inspired by STS literature, we did not assume that we knew what a high-profile case was, nor did we look for a definition of what it might be. Rather we asked: what is a high-profile case? One of STS’ major contributions is its insistence on practices as important sites to learn about how objects or subjects come about. STS has also alerted us to the mundane material aspect of objects and subjects. In this vein, we have followed the Vaatstra case around so as to unravel the stuff that the high-profile case is made of. We did not take the contours of the case at face value, but rather kept an open eye for ‘odd’ elements that persisted and kept being reported on, as if in the shadow of the Vaatstra case, such as road lighting, or the knife. It was such elements that helped us to open up the case for interrogation and to demonstrate how, depending on the concerns it was drawn into, it kept changing shape. The changes could not simply be explained by the context of the case, such as the village, Dutch immigration policy, etc., because as we have seen it was rather the other way around. It was the identity of the village, Dutch legislation, forensic infrastructure, among other things, that were at stake and were subject to change (M’charek, 2016). The Vaatstra case is an inherently indeterminate object. Its unruliness took the shape of a fire-like pattern of destruction and creation, generating and depending on absences and presences. This means that the high-profileness of a case neither inheres in the specificities of the crime committed, nor in the (media) attention it receives. It rather lies in its capacity to shift and change and to engage other societal concerns.

## Notes

1. Roland Bal’s study (2005) of another Dutch high-profile case, the ballpoint murder, examines the boundary work between credible and non-credible expertise in the courtroom. The case, including how it appeared in the media, allowed Bal to make this boundary work explicit.

2. Documentary: ‘Een nacht van 800 dagen’ (*Omrop Fryslan*), broadcast June 2001.

3. The Dutch term *Zinloos Geweld* (‘senseless violence’) resonates with the social concern over ‘random violence’ in the United States (Stengs, 2007: 159).

4. In its form and organization, the silent march resonates with the ‘white marches’ in Belgium (Boutellier, 2005).

5. The term allochthonous (lit. ‘not from the soil’) is used to indicate those of non-Dutch birth or ancestry, whereas autochthonous (lit. ‘from the soil’) is used for those of Dutch birth and ancestry. The terms came to be used in public policy and national statistics, and have since become mainstream in public discourse (Essed and Nimako, 2006; Geschiere, 2009).

6. In her research on the notion of senseless violence in the Netherlands, Stengs points to cases in which the perception that ‘the division between “good” and “evil” parallels the ethnic distinction between “we” (i.e. autochthonous Dutch and victims) and “them” (i.e. Moroccans and perpetrators)’ was more explicitly expressed (Stengs, 2007: 177).

7. See crime watch show *Peter R de Vries* (2003).

8. Within the tradition of Actor Network Theory enrolment is understood as the definition and distribution of roles as an effect of multilateral negotiations between both human and non-human actors (Callon, 1984).

9. See also Rigter (2002), who studied the protest against the permanent asylum seeker centre in Kollum as a social movement.

10. Sociologist and politician Pim Fortuyn became known as the party leader of ‘Leefbaar Nederland’ in 2001 and later of his own party ‘Lijst Pim Fortuyn’. He was critical of Islam and argued for a stricter asylum policy. He was shot to death in 2002 by an animal rights activist.

11. Showing head shots and full names of suspects on television was a controversial act. As the Public Prosecutor commented: ‘This could be in conflict with journalistic ethics and the protection of individual privacy’ (*Trouw*, 31 August 1999).

12. Greer and McLaughlin (2012: 3) define ‘trial by media’ as ‘a market-driven form of multi-dimensional, interactive, populist justice in which individuals are exposed, tried, judged and sentenced in the “court of public opinion”’.

13. See Toom (2011) and M’charek (2008) for a more detailed account of the development of Dutch legislation regarding forensic DNA.

14. See Toom and M’charek (2011) for a discussion of how different applications of forensic DNA technologies may conflict with the presumption of innocence.

15. The Dutch word ‘*buurt*’ may refer to ‘vicinity’ as well as ‘neighbourhood’, which strengthened the association with ‘closeness’.

16. The widely explored notion of the ‘CSI effect’ (e.g. Kruse, 2010; Machado and Santos, 2011; Mopas, 2007) thus only partially connected to the Vaatstra case, as the reality of the incrimination of asylum seekers here interfered with the belief in forensic science as a ‘super science’ that is always ‘accurate and infallible’ (Machado and Santos, 2011: 306).

17. The Dutch word used here is ‘*honkvast*’, which more literally translates into ‘fixed to base’ and resonates with ‘stay-at-home’.
